# Sex-Specific Differences in Agonistic Behaviour, Sound Production and Auditory Sensitivity in the Callichthyid Armoured Catfish *Megalechis thoracata*

**DOI:** 10.1371/journal.pone.0121219

**Published:** 2015-03-16

**Authors:** Oliwia Hadjiaghai, Friedrich Ladich

**Affiliations:** Department of Behavioural Biology, University of Vienna, Vienna, Austria; University of Auckland, NEW ZEALAND

## Abstract

**Background:**

Data on sex-specific differences in sound production, acoustic behaviour and hearing abilities in fishes are rare. Representatives of numerous catfish families are known to produce sounds in agonistic contexts (intraspecific aggression and interspecific disturbance situations) using their pectoral fins. The present study investigates differences in agonistic behaviour, sound production and hearing abilities in males and females of a callichthyid catfish.

**Methodology/Principal Findings:**

Eight males and nine females of the armoured catfish *Megalechis thoracata* were investigated. Agonistic behaviour displayed during male-male and female-female dyadic contests and sounds emitted were recorded, sound characteristics analysed and hearing thresholds measured using the auditory evoked potential (AEP) recording technique. Male pectoral spines were on average 1.7-fold longer than those of same-sized females. Visual and acoustic threat displays differed between sexes. Males produced low-frequency harmonic barks at longer distances and thumps at close distances, whereas females emitted broad-band pulsed crackles when close to each other. Female aggressive sounds were significantly shorter than those of males (167 ms versus 219 to 240 ms) and of higher dominant frequency (562 Hz versus 132 to 403 Hz). Sound duration and sound level were positively correlated with body and pectoral spine length, but dominant frequency was inversely correlated only to spine length. Both sexes showed a similar U-shaped hearing curve with lowest thresholds between 0.2 and 1 kHz and a drop in sensitivity above 1 kHz. The main energies of sounds were located at the most sensitive frequencies.

**Conclusions/Significance:**

Current data demonstrate that both male and female *M*. *thoracata* produce aggressive sounds, but the behavioural contexts and sound characteristics differ between sexes. Sexes do not differ in hearing, but it remains to be clarified if this is a general pattern among fish. This is the first study to describe sex-specific differences in agonistic behaviour in fishes.

## Introduction

A wealth of information is available on sound-generating mechanisms, sound production during agonistic and reproductive behaviour, and hearing in fishes (for reviews see [1–5]). Despite this knowledge, data on sex-specific differences in sonic organs, sound production and in hearing are very limited [6].

Studies in several families such as Gadidae, Ophidiidae, Batrachoididae, Osphronemidae and Sciaenidae described sexual dimorphism of sound-generating structures [7–11]. Pectoral as well as swimbladder structures for sound production are typically larger in males than females. Kratochvil [12] found that pectoral sonic muscles and tendons were larger in male than female gouramis (genus *Trichopsis*, family Osphronemidae), and Pruzsinszky and Ladich [13] revealed that pectoral fin spines of male peppered corydoras *Corydoras paleatus* were relatively longer than those of females. Similarly, swimbladder sonic muscles were typically larger in males than females in representatives of the families Batrachoididae (e.g. oyster toadfish *Opsanus tau* and midshipman *Porichthys notatus;* [14–15]), Sciaenidae (e.g. Japanese croaker *Argyrosomus japonicus*, [16]) and Gadidae (e.g. haddock *Melanogrammus aeglefinus* [7]). Kéver et al. [17] recently showed that male cusk-eels *Ophidion rochei* additionally possess larger drumming muscles and a rocker bone on the swimbladder, which is lacking in females.

Investigations on agonistic behaviour often revealed that both sexes generate sounds and that sex-specific differences in agonistic sounds are rather small [18–21] (The term “agonistic behaviour” used here includes intraspecific aggressive interactions as well as interspecific disturbance situations). The agonistic context differs considerably from the reproductive context, where except for one species (*Trichopsis vittata* [20]) only males emit advertisement or courtship calls. Acoustic displays always occur in combination with visual displays during agonistic interactions, which generally start when opponents are detected visually. Brawn [22] mentioned that in non-spawning periods both sexes of the Atlantic cod *Gadus morhua* (formerly *G*. *callarias*) produce grunts that help to intimidate other cod. Myrberg et al. [18] noted that males and females of the jewelfish *Hemichromis bimaculatus* produced pulsed br-r-r sounds before attacking an intruder. In the flier cichlid *Archocentrus centrarchus* (formerly *Cichlasoma centrarchus*), female attacks on males are often accompanied by low-frequency growls [23]. Ladich [24] reported that in the European river bullhead *Cottus gobio* both sexes produced two sound types while defending their territories and that females vocalized less than males. Sex-specific differences in sound characteristics uttered in the same contexts were rarely described. Male pinhead pearlfish *Carapus boraborensis* emitted shorter pulses than females [21]. Both male and female *T*. *vittata* produced long, high-intensity croaking sounds during agonistic encounters, which did not differ in sound characteristics except sound levels [20]. Simões et al. [25] found that the male agonistic sounds of the zebra mbuna *Maylandia zebra* (formerly *Pseudotropheus zebra*) lasted longer and consisted of more pulses than those of females. Oliveira et al. [26] showed a major similarity in feeding and courtship clicks as well as distress growls in both sexes of the longsnout seahorse *Hippocampus reidi*.

A previous study on the callichthyid armoured catfish *Megalechis thoracata* (formerly *Hoplosternum thoracatum*) showed that, corresponding to the behavioural contexts, males and females uttered different types of pectoral sounds [27]. This differs considerably from investigations on the callichthyid *C*. *paleatus*, in which males produced trains of sounds during dyadic contests, whereas no stridulation sounds could be recorded from females during social interactions [13, 28].

All otophysans including catfishes possess a Weberian apparatus, which connects the swimbladder to the inner ears and distinctly improves their hearing ability (for reviews see [29–30]). Sex-specific differences in hearing sensitivities were found in the round goby *Neogobious melanostomus* [31] and in the African cichlid fish *Astatotilapia burtoni* depending on the animal’s internal hormonal state [32] but not in other species (*C*. *paleatus* [33]; Atlantic molly *Poecilia mexicana* [34]; Hawaiian sergeant damselfish *Abudefduf abdominalis* [35]).

Acoustic communication is defined as transmission of information by a sender to a receiver with benefits for the receiver or mutual potential benefits for both [36]. Accordingly, a match of spectral contents of sounds and best hearing sensitivity of the intended receiver, and vice versa, should be preferred by natural selection [33, 35]. Auditory sensitivity was found to match the characteristics of sounds produced in the frequency domain in some species [35, 37–41], but mismatch was observed in others [33, 42].

The aims of the present study were to investigate sex-specific differences in (1) sound-generating mechanisms, (2) agonistic behaviour, (3) sounds produced and their characteristics, (4) the auditory abilities, and finally (5) to determine if the dominant frequencies of sounds correlate with the best hearing sensitivity in the callichthyid catfish *M*. *thoracata*.

## Material and Methods

### Ethics statement

Experiments were performed with permission of the Austrian Federal Ministry of Science and Research (Bundesministerium für Wissenschaft und Forschung, GZ 66.006/0023-II/10b/2008). The permission covered all experimental procedures. No animal was sacrificed during this study. According to the Austrian Law on Experiments in Animals (Tierversuchsgesetz 1989/2005) an institutional animal care and use committee (or ethics committee) was not mandatory and did not exist at the University of Vienna when experiments were carried out.

### Animals

Seventeen specimens of *M*. *thoracata* were used for the study: seven subadult males (93–104 mm total length, TL, 12.2–19.1 g body mass), nine subadult females (84–102 mm, 9.8–17.1 g) and one adult male (126 mm, 32.9 g). Fish were obtained from a local pet shop and a fish farmer. We assume that 16 of the 17 fish were subadult because they were one year old during our experiments and Mayr [27] stated that first reproductive behaviour in this species is shown at the age of two years. Moreover, we observed no reproductive behaviour in the present study when sexes were kept together in community tanks. This species is a bottom-dwelling fish from slowly flowing rivers, pools, drainage ditches and swampy areas in South America [43].

The sex of the fish was determined by inspecting the genital papillae, the distance between coracoids and the size of pectoral fins. Males possess genital papillae, a narrower gap between coracoids and longer, thicker and orange pectoral spines [43]. Due to the individually spotted body pattern, the subjects could be easily distinguished from each other.

Fish were kept in three community tanks (110 x 30 x 55 cm) whose bottom was covered with fine sand and which were similarly equipped with half flower pots, tubes, plants and roots as shelters. The tank of the adult male measured 90 x 30 x 30 cm. Four males and four females and three males and five females, respectively, were kept together. A 12:12 hour light:dark cycle was provided and the water temperature was kept at 25 ± 1°C. The aquaria were filtered by external filters in order to reduce noise. Fish were fed frozen chironomid larvae and occasionally artificial food (flakes and tablets) five to six times a week.

### Morphological measurements

After behaviour and sound recordings, body mass, total length and length of the pectoral spine of each contestant were measured. The pectoral spine length (PSL) was measured from the juncture of the spine with the outer body surface to its tip using digital callipers. The relative pectoral spine length (rPSL) was calculated using the formula rPSL = PSL/TL, where TL is the total length.

### Recording of behaviour and sounds

All agonistic experiments were performed from August to November 2011. Fish were kept for three months in holding tanks before the start of behavioural experiments. The video and sound recordings were carried out in a walk-in soundproof room, which was constructed as a Faraday cage. The experiments were conducted in a test tank (70 x 40 x 35 cm). The resonant frequency of the tank is 3042 Hz according to Akamatsu et al. [44]. The water temperature was maintained at 25 ± 1°C. The test tank was placed on a table that rested on a vibration-isolated concrete plate.

The behaviour and acoustic signals were recorded using a hydrophone (Brüel & Kjaer 8101, sensitivity −184 dB re 1 V μPa^−1^), which was connected to a power supply (Brüel & Kjaer 2804) and placed in the centre of the aquarium close to the back wall. Both the hydrophone and video camera (Sony CCD-VX1E) were connected to a HiFi S-VHS video cassette recorder (JVC HR-S4700 EG/E). HiFi audio and S-video signals were stored simultaneously on S-VHS HiFi videotapes (FUJIFILM Super VHS PRO SE-240). Sound pressure levels (RMS Fast time weighting, Linear frequency weighting) were measured in parallel with the sound recordings using a sound level meter (Brüel & Kjaer Mediator 2238) connected to the power supply. The observer was always hidden behind a curtain during recordings. External filters of the test tank were switched off before the start of experiments.

In order to reduce prior dominance experience, fish were isolated for five days in isolation tanks (50 x 30 x 27 cm) and then for two more days in the test tank, which was divided by a non-transparent plastic sheet. Fish in each dyadic pairing came from different holding tanks. Both halves of the test tanks were equipped with a half flower pot as shelter, plants and a sand bottom. On day eight, the separating sheet between two males or two females was removed and video and sound recordings started. Dyadic encounters were recorded for thirty minutes and fish were separated after this period. Fish were observed for an additional ten minutes after separation to determine if sounds were produced without visual contact. Due to the small number of individuals and the fact that acoustic displays occurred rarely, animals were used repeatedly in order to achieve a higher number of agonistic contests and thus sound recordings. The same fish, however, were never paired twice.

As the intention of this study was to record acoustic displays during agonistic behaviour dyadic interactions were not allowed to proceed to a phase, where biting might have occurred. No biting or injuries were observed in any of the experiments.

### Analysis of behaviour and sounds

Each sound was digitized using a sampling rate of 11 kHz (16 bit resolution) and analysed using Cool Edit 2000 (Syntrillium Software Corporation, Phoenix, USA) and ST^x^ Soundtools 3.7.8. (Institute of Sound Research at the Austrian Academy of Sciences, Vienna). Only sounds with a good signal-to-noise ratio were analysed. The following acoustic variables were measured from sounds recorded during dyadic contests:

Sound duration (SD): the total length between the onset and the end of a single call or a series of sounds. Dominant frequency (DF): the frequency with the highest amplitude within a power spectrum. The dominant frequency of sounds was determined from cepstrum-smoothed power spectra. Harmonic sounds are characterized by several regularly spaced peaks, in which the frequencies of the harmonic peaks are multiples of that of the fundamental frequency. The harmonic content of a sound was controlled by overlaying a harmonic grid.

Sound pressure level (SPL): measured in dB re 1μPa (RMS Fast, Linear weighting). In order to compensate the varying distances of vocalizing fish to the hydrophone, a correction factor was calculated. Therefore, the test tank was divided into 21 sectors (each measuring 10 x 10 cm) by using a grid applied on the front glass of the aquarium. The sector in which a fish emitted sounds was noted. Because sounds of *M*. *thoracata* were of low energy, pink noise was chosen for calculating a correction factor. Short tone bursts were played back at a constant SPL from a small loudspeaker (Fuji 7G06, 8 Ohm, 0.8 W), in each of the 21 sectors, and the SPLs were noted. The relative difference of the SPL measured in the sector nearest to the hydrophone (3 cm away) and the other sectors were calculated and added to the SPL values of fish sounds measured before. Thus, a distance-independent absolute SPL value could be determined for each sound emission.

The aim of the behavioural analyses was to describe all behavioural patterns (elements) occurring during the male-male and female-female agonistic contests. All behavioural patterns shown during a total of 24 agonistic encounters were classified according to the description presented in Table 1. The number of acoustic signals and visual displays such as attack, circling and head nodding was counted for each experiment and individual.

**Table 1:** Description of the behavioural repertoire shown during dyadic male-male and female-female interactions of *M. thoracata*.

Behavioural pattern	Brief Description
**Non-aggressive behaviour**	
Air gulping (m, f)	Air intake at the water surface.
Comfort (m, f)	Remaining in stationary position on the bottom, alone or with another fish.
Swimming (m, f)	Directed and non-directed locomotion of individuals.
Digging (m, f)	Picking up sand with mouth and spitting it out immediately.
**Aggressive behaviour**	
Approach (m, f)	Fish swam in the direction of another individual.
Attack (m, f)	Fin displaying or approaching another fish, stopping suddenly before forcefully hitting the other with its tail.
Chase (m, f)	One fish rapidly pursued another individual.
Circling (f)	Two females swam in an anti-parallel (head-to-tail) orientation.
Fin beating (m)	Two male opponents performed undulating movements with their erected fins during fin displays.
Fin display (m)	Two males moved towards each other, in a parallel, anti-parallel position or various angles spreading all their fins. Additionally, at high intensities the caudal part of the body was erected in a distinct angle.
Fleeing (m, f)	Continued escape reaction in response to a chase. Fish swam rapidly away from the aggressor.
Head nodding (f)	Females showed serial vertical up and down movements of their heads in different positions to each other.
Jerk (m)	Males moved their heads rapidly away and towards the opponent while bending their body C-like.
Sneaking (f)	One female following the opponent by a snake-like movement on the bottom.
Strike (f)	One female swam rapidly toward the opponent while vocalizing.

Definitions followed Mayr [27] except for fin beating, jerks and sneaking. m—males, f—females.

### Auditory sensitivity measurements

Auditory sensitivity was determined using the non-invasive auditory evoked potential (AEP) recording technique, originally reported by Kenyon et al. [45] and modified by Wysocki and Ladich [46–47].

Test animals were mildly immobilized by injecting intramuscularly Flaxedil (gallamine triethiodide; Sigma-Aldrich Handels GmbH, Vienna, Austria). The dosage applied was 11.42–15.13 μg g^−1^ for males and 10.79–14.52 μg g^−1^ for females, thus enabling the immobilized fish to produce slight opercular movements. All auditory measurements were carried out in an oval plastic tub (diameter 45 x 33 cm, water depth 12 cm, 1 cm layer of sand). The tub was positioned on an air table (TMC Micro-g 63–540, Technical Manufacturing Corporation, Peabody, MA, USA), which rested on a vibration-isolated concrete plate. The entire experimental setup was enclosed in a walk-in soundproof chamber (interior dimensions: 3.2 x 3.2 x 2.4 m), which was constructed as a Faraday cage.

The subjects were positioned in the centre of the tub, so that the nape of the head was at the water surface. Respiration pipettes were inserted into the animal’s mouth. Respiration was achieved through a simple, temperature-controlled (25 ± 1°C), gravity-fed water circulation system. A small piece of tissue paper was placed on the fish head to keep it moist and ensure proper contact of electrodes during experiments. The AEPs were recorded using silver wire electrodes (diameter 0.38 mm), which were pressed firmly against the fish’s skin. The recording electrode was placed at the brainstem region and the reference electrode cranially between the nares. Shielded electrode leads were attached to the differential input of an a.c. preamplifier (Grass P-55, Grass Instruments, West Warwick, RI, USA; gain 100x, high-pass at 30 Hz, low-pass at 1 kHz). A ground wire was placed underwater near the subject.

Sound stimuli presentation and AEP waveform recording were achieved using a Tucker-Davis Technologies (Gainesville, FL, USA) modular rack-mount system (TDT System 3) controlled by a PC containing a TDT digital signal processing board and running TDT BioSig RP software. A dual-cone speaker (Wharfedale Pro Twin 8, frequency response: 65 Hz—20 kHz ± 3 dB), mounted 0.5 m above the fish in the air, was used to present tone stimuli during testing. Acoustic stimuli consisted of tone bursts presented at a repetition rate of 21 s^−1^. Hearing thresholds were determined at the following frequencies: 0.1, 0.2, 0.3, 0.5, 1, 2, 3 and 4 kHz, always presented in random order. A hydrophone (Brüel & Kjaer 8101; frequency range 1 Hz—80 kHz ± 2 dB; voltage sensitivity −184 dB re 1 V μPa^−1^) was positioned on the right side of the fish (approximately 2 cm away) to determine absolute stimulus SPLs underwater in close proximity to the subjects. To enhance the hydrophone signal (1000 x), a second custom-built preamplifier was used.

For each test condition, the stimuli were presented at opposite polarities (180° phase shifted) and the corresponding AEPs were averaged in order to eliminate stimulus artefacts. At SPLs close to the threshold, this procedure was performed at least twice and the AEP traces were overlaid to examine if they were repeatable. The SPL values of tone burst stimuli were reduced in 4 dB steps. By overlaying replicate traces, the lowest SPL in which an identifiable and repeatable AEP trace could be obtained was regarded as threshold. The method developed by Kenyon et al. [45] gives auditory thresholds very similar to behavioural thresholds (for discussion see [4, 48]).

### Statistical analysis

All data were tested for normal distribution using the Kolmogorov-Smirnov-test and, when data were normally distributed, parametric statistical tests were applied. Means of sound characteristics (duration, dominant frequency and SPL) were calculated for each individual for further analyses. Relationships between morphological variables (total length, pectoral spine length, relative pectoral spine length) and sound characteristics were determined by Pearson’s correlation coefficients and linear regressions. Correlations were calculated by including all males which produced a certain sound type, and all females. Thus, a correlation was calculated for all males which produced barks, and all females, as well as a correlation for all males which produced thumps, and all females. Total length was chosen to allow comparison with prior studies. Only minor differences were found in correlation coefficients between total length and standard length in the current study.

Additionally, a one-way analysis of variance (ANOVA) was performed, followed by a Bonferroni post-hoc test, in order to determine differences in sound characteristics of each aggressive sound type. Differences in relative pectoral spine length between sexes were tested using a paired T-test. A total of 244 sounds were analysed.

Mean hearing thresholds were determined for both sexes at each frequency. Thresholds obtained for males (N = 6) and females (N = 6) were compared by a two-way ANOVA using a general linear model, where one factor was sex and the other was frequency. The sex factor alone should reveal differences in sensitivity between sexes and, combined with the frequency factor, if different tendencies exist at different frequencies of the audiograms.

All statistical tests were conducted using PASW 18.0 (SPSS Inc., Chicago, USA). The significance level was set at p ≤ 0.05.

## Results

### Pectoral fins

**Fig 1:**
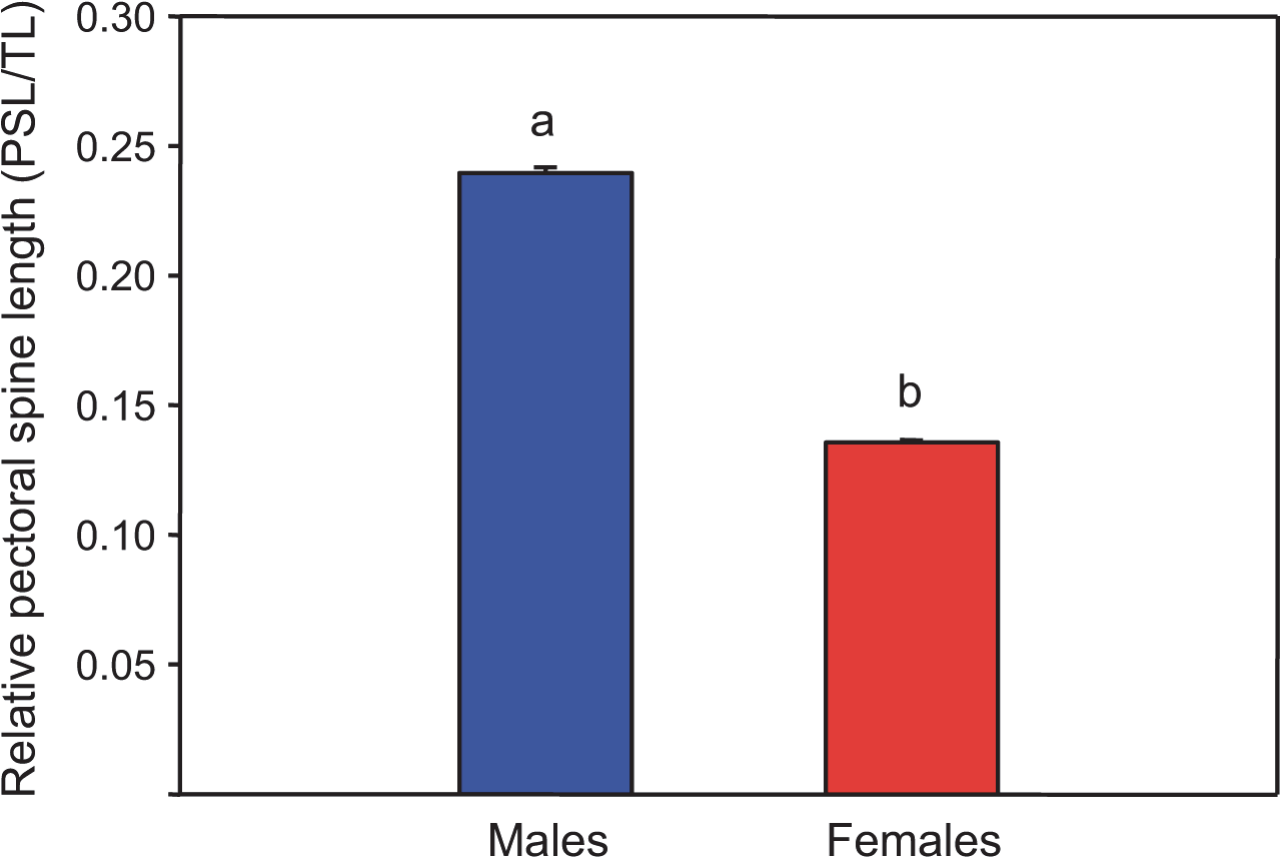
Mean (+ SE) relative pectoral spine length of male and female *Megalechis thoracata*. Different letters indicate statistically significant differences between sexes. PSL—total length of pectoral spine, TL—total fish length.

*Megalechis thoracata* produced sounds by vibrating pectoral fins. The first pectoral fin rays were orange in males in contrast to females. Pectoral spines were longer and thicker in males. The relative length of pectoral spines (pectoral spine length/total length) was on overage 1.7-fold higher in males than females (T-Test, t = 44.27, df = 15, p ≤ 0.01) (Fig. 1). Microscopic dissection and inspection of an alcohol-preserved adult male revealed that *M. thoracata* possessed a pair of tiny bony encapsulated bladders lacking any drumming muscles.

### Agonistic behaviour

Aggressive interactions usually started after removal of the separating sheet and as soon as opponents detected each other visually. The agonistic behavioural sequences were interrupted by air gulping, digging, resting close to each other or withdrawal into the shelter. For the description of behavioural patterns observed during thirteen male-male and eleven female-female encounters see Table 1.

### Male-male contests

Generally, one or both males started to approach each other and erected their fins (threatening fin display). Occasionally, this posture was followed by fin beating, which could last for several seconds. Head jerking followed and was accompanied by the production of two sound types, namely barks or thumps, indicating high levels of aggression. Jerking could occur without sound emission, in which case maximally three jerks were observed.

**Fig 2:**
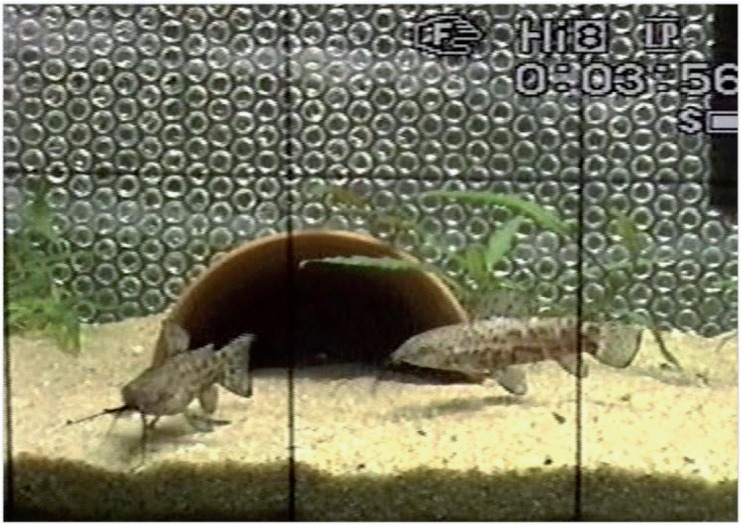
Screen shot of a video recording showing two males in an agonistic contest. The illustration shows the right male approaching the opponent, shortly before uttering a bark.

In 12 out of 13 encounters (92%), barks and thumps were produced during aggressive interactions. Furthermore, in 10 out of 13 contests (78%), sound emission occurred in both contestants. Barks were emitted at various distances from a few centimetres up to a maximum of 40 cm away from the opponent. Before abduction of pectoral fins, a single adduction was observed. Barks were often produced during approaching and swimming (Fig. 2). The individual producing a sound mostly swam away and then again approached the conspecific. The latter could react by fin displays or moving away from the other.

**Fig 3:**
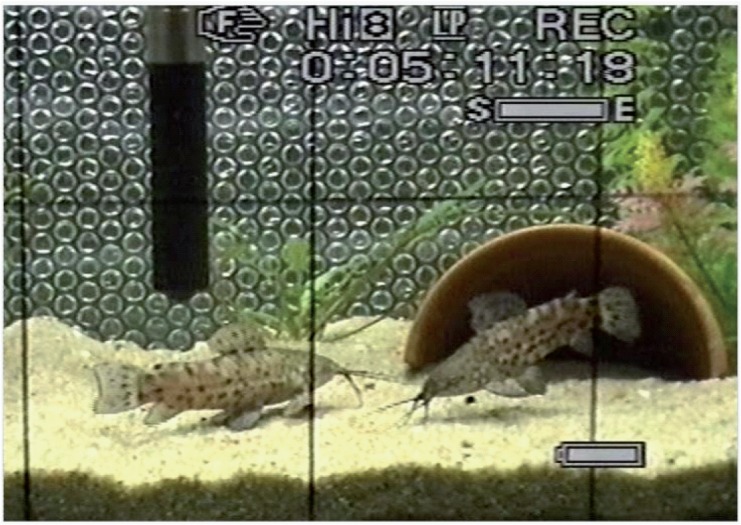
Screen shot of video recording during a male-male aggressive interaction. This screen shot shows the typically threatening fin display with fins spread and the caudal part of the right male erected in a distinct angle. The right fish produced a thump in this oblique anti-parallel position relative to the conspecific.

In contrast to barks, thumps occurred only in direct proximity (within one body length) to the opponent. Typically, opponents showed fin displays in a parallel, anti-parallel position or at various angles to each other (Fig. 3), or swam close and emitted thumps. Typically, thumps were produced in an oblique position towards the opponent. Opponents responded by producing a thump, by attacking or by fleeing.

During all experiments, we counted a total of 40 attacks, often preceded by fin display and fin beating. Attacks were mainly performed by sound producers, occasionally accompanied by thumps. Besides these behavioural elements, chasing was observed. During chasing, barks were emitted by the pursuer.

In five out of seven experiments, barks were even emitted after separating both fishes by the plastic sheet, i.e. after they lost visual contact. Barks were recorded mainly in males, which produced numerous sounds during the dyadic contests. Just in one case did both opponents emit barks. After separation by the plastic sheet, males generated barks during swimming or when approaching the separating sheet.

### Female-female contests

**Fig 4:**
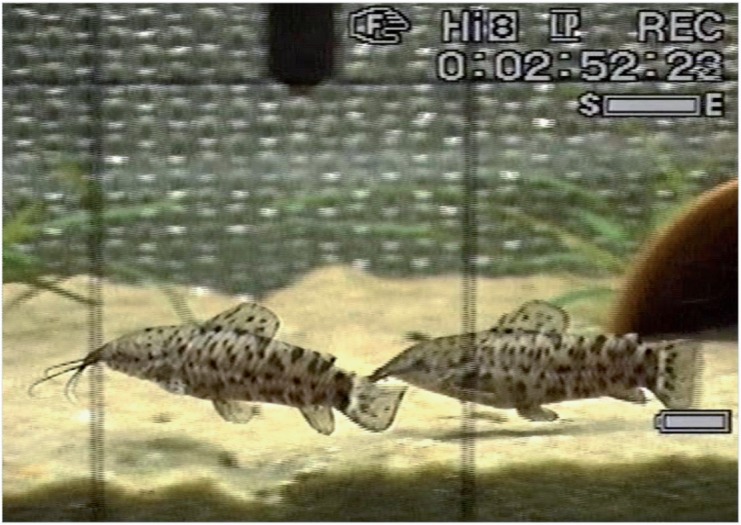
Screen shot of video recording illustrating a female-female agonistic encounter. The right female emitted a series of crackles while pursuing the other female.

Interactions started when one female approached the other, in a few cases by sneaking movements (Table 1) close to the bottom. This sneaking behaviour toward the opponent was occasionally accompanied by the emission of crackles. Sounds were recorded in all female-female experiments. In four out of eleven contests, both females vocalized during agonistic interactions. Sounds were produced close to the opponent (within one to two body lengths) (Fig. 4) and were generated by rapid pectoral fin movements. Shortly before uttering a crackle, the vocalizing fish may strike towards the opponent. Crackles were most frequently emitted when one female chased the other. Swimming after each other or pursuing could continue into circling behaviour (Table 1), which lasted up to 3 s. Sounds were uttered before, during or at the end of circling.

Head nodding was either performed by one female or simultaneously by both. Two to 18 head nods per individual were observed within a series. We counted a total of 736 head nods during eleven agonistic interactions, and the vocalizing female exhibited a higher rate. This visual threatening display could be elicited by the movement of one fish, when swimming by or when above the other, before and after circling, and when resting close to each other. Furthermore, interactions were characterized by a high rate of body contacts such as touching with barbels. Attacks occurred only four times: three were accompanied by crackles and one was exhibited shortly after head nodding.

In contrast to male-male encounters, in which both males vocalized, crackles were mainly produced by one female, seldom by both. Female-female contests were characterized by circling behaviour and the lack of jerks. Head nodding could not be observed in any male-male interaction. While males beat their fins during fin displays, females did it during circling. Females undulated only their dorsal and caudal fins, whereas in males all fins were involved. Furthermore, females attacked less frequently than males, indicating a lower level of aggressivity.

### Vocalizations

Three types of sounds were recorded during dyadic encounters. Males produced barks and thumps, and females emitted crackles. Barks and thumps were recorded in seven out of eight males and crackles in eight out of nine females. The fish produced 376 barks, 66 thumps and 739 crackles during 24 interactions. Acoustic signals were not audible to human listeners. Video analysis revealed that barks were produced during abduction of the pectoral fin. We could not unequivocally determine the movement of the pectoral fins (adduction and/or abduction) during the emission of thumps and crackles.

### Male agonistic sounds

**Fig 5:**
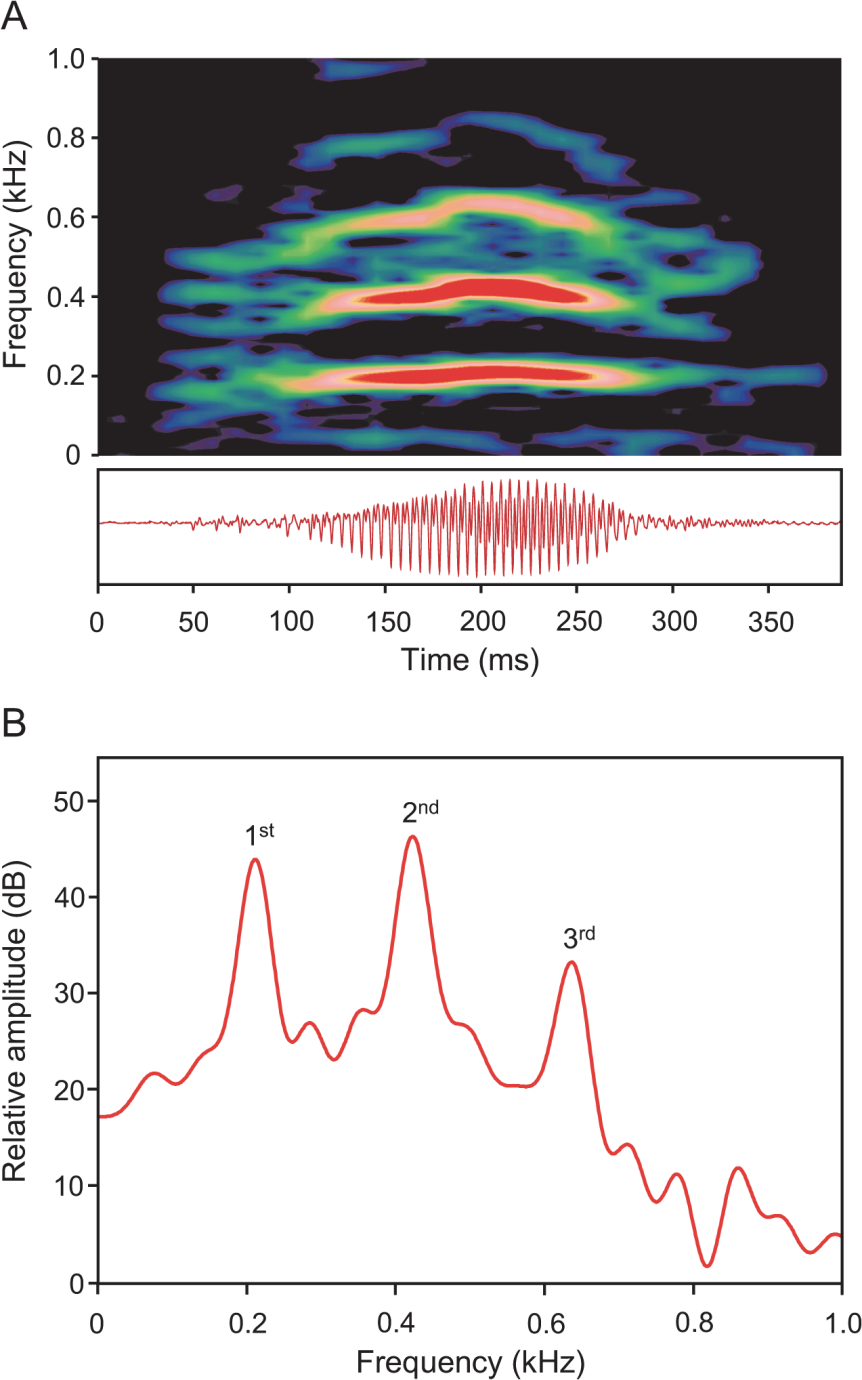
(A) Sonagram (top) and oscillogram (below) and (B) cepstrum-smoothed power spectrum of a monopartite bark of a male *M. thoracata* produced during an aggressive encounter. The spectrum shows three harmonics (1st, 2nd, 3rd) with the highest energy found in the second harmonic. Sampling rate 22 kHz. Hanning filter, overlap 75%, (A) filter bandwidth 20 Hz, (B) filter bandwidth 1 Hz, number of coefficients 350.

**Fig 6:**
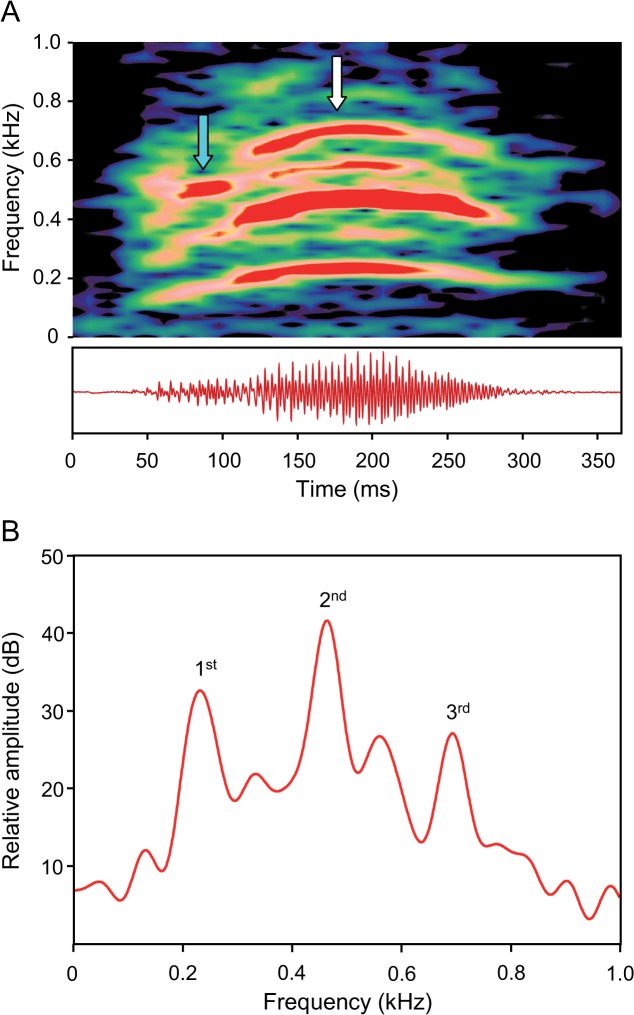
(A) Sonagram (top) and oscillogram (below) and (B) cepstrum-smoothed power spectrum of the bipartite bark of a male *M. thoracata*. The spectrum reveals three harmonics (1st, 2nd, 3rd) within the second sound part (white arrow) with the highest energy found in the second harmonic. The oscillogram shows lower amplitude in the first part (light blue arrow). Sampling rate 11 kHz. Hanning filter, overlap 75%, (A) filter bandwidth 20 Hz, (B) filter bandwidth 1 Hz, number of coefficients 170.

Barks were low-frequency harmonic sounds showing frequency modulation; they only occurred singly. They consisted of one to three parts and were therefore classified as being mono-, bi- or tripartite (Fig. 5A, B). The most common sound structure was bipartite, in which the second part had a higher amplitude than the first part (Fig. 6A, B). Sound duration ranged from 159 to 317 ms, and the SPL ranged from 101.2 to 125 dB re 1 μPa at a distance of 3 cm. The main energies were found in the first, second or third harmonic. The dominant frequency of bipartite barks varied in the first part from 110 to 600 Hz, and in the second from 170 to 730 Hz.

**Fig 7:**
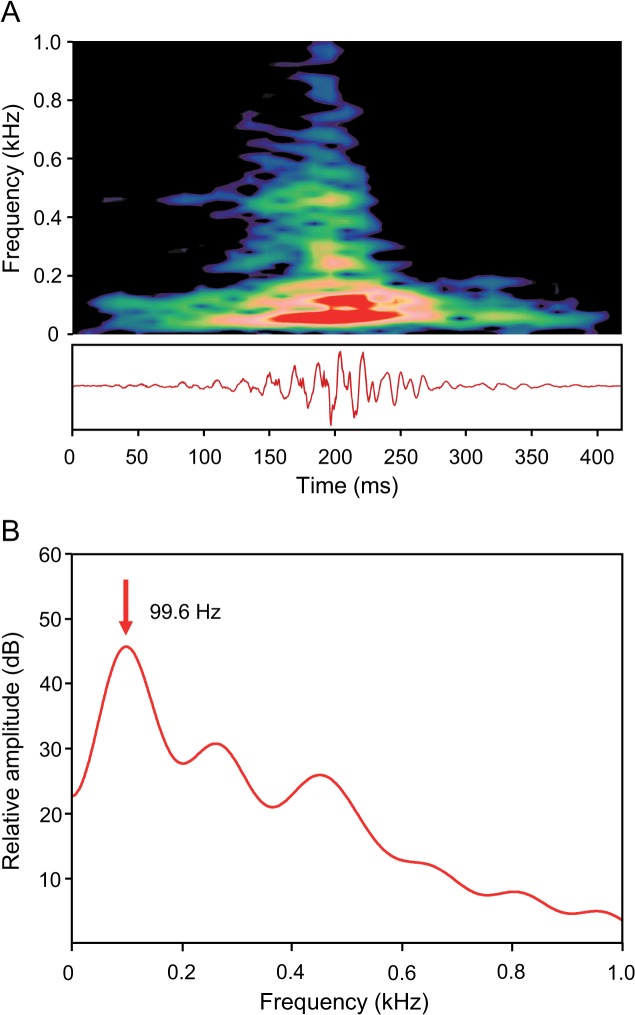
(A) Sonagram (top) and oscillogram (below) and (B) cepstrum-smoothed power spectrum of a monopartite thump of a male *M. thoracata* uttered in an agonistic context. The dominant frequency is indicated in (B). Sampling rate 11 kHz. Hanning filter, overlap 75%, (A) filter bandwidth 20 Hz, (B) filter bandwidth 1 Hz, number of coefficients 80.

Thumps were produced singly and showed no harmonic structure. They were mostly mono-, seldom bipartite (Fig. 7A, B). Bipartite thumps were recorded only in the one examined adult subject. Sound duration ranged from 116 to 446 ms and the SPL ranged from 107.07 to 137.5 dB re 1 μPa. The dominant frequency varied from 70 to 210 Hz.

### Female agonistic sounds

**Fig 8:**
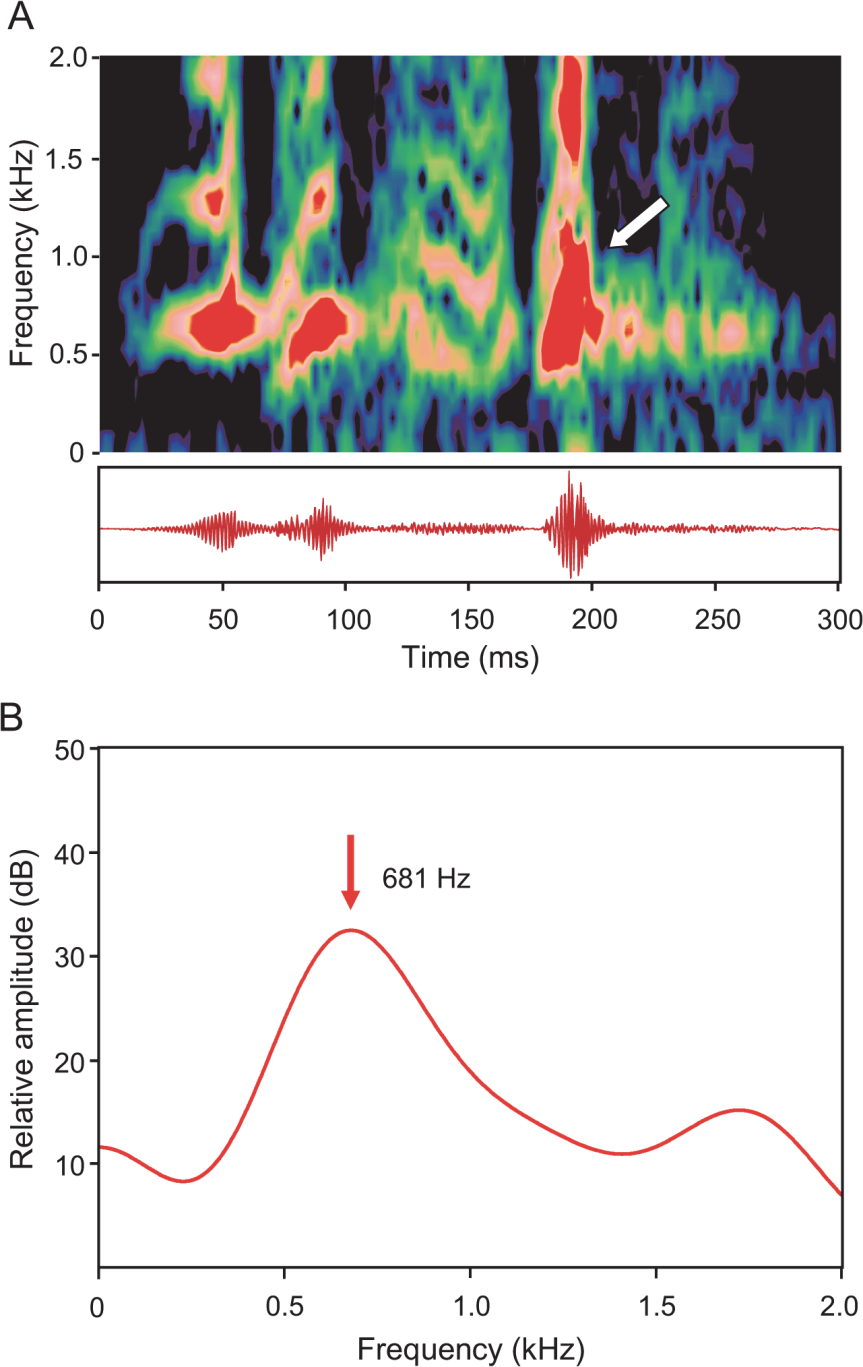
(A) Sonagram (top) and oscillogram (below) and (B) cepstrum-smoothed power spectrum of crackles of a female *M. thoracata* produced in an agonistic context. The oscillogram shows five sound elements. The white arrow indicates the main element, followed by a train of pulses. The dominant frequency is indicated in the cepstrum-smoothed power spectrum in (B). Sampling rate 11 kHz. Hanning filter, overlap 75%, (A) filter bandwidth 100 Hz, (B) filter bandwidth 1 Hz, number of coefficients 20.

Female crackles differed from male sounds in their complex structure and frequency content. They were of higher frequency and always consisted of series of sound elements (Fig. 8A, B). Crackles were built up mostly by four sound elements (range: 2–8 elements). These series were characterized by a main element featuring the highest peak-to-peak-amplitude and several elements of lower amplitude before and after the main element (Fig. 8A). Elements could be separated by intervals from each other and could consist of a substructure such as a train of pulses (= one element).

The duration of crackles ranged from 81 to 394 ms. Single elements (including train of pulses) ranged from 5.6 up to 192.9 ms and main elements varied from 15.3 to 57.6 ms in duration. SPLs were between 101.5 and 128.1 dB re 1 μPa. Dominant frequencies of crackles varied from 370 to 830 Hz.

### Comparison between sound types

**Fig 9:**
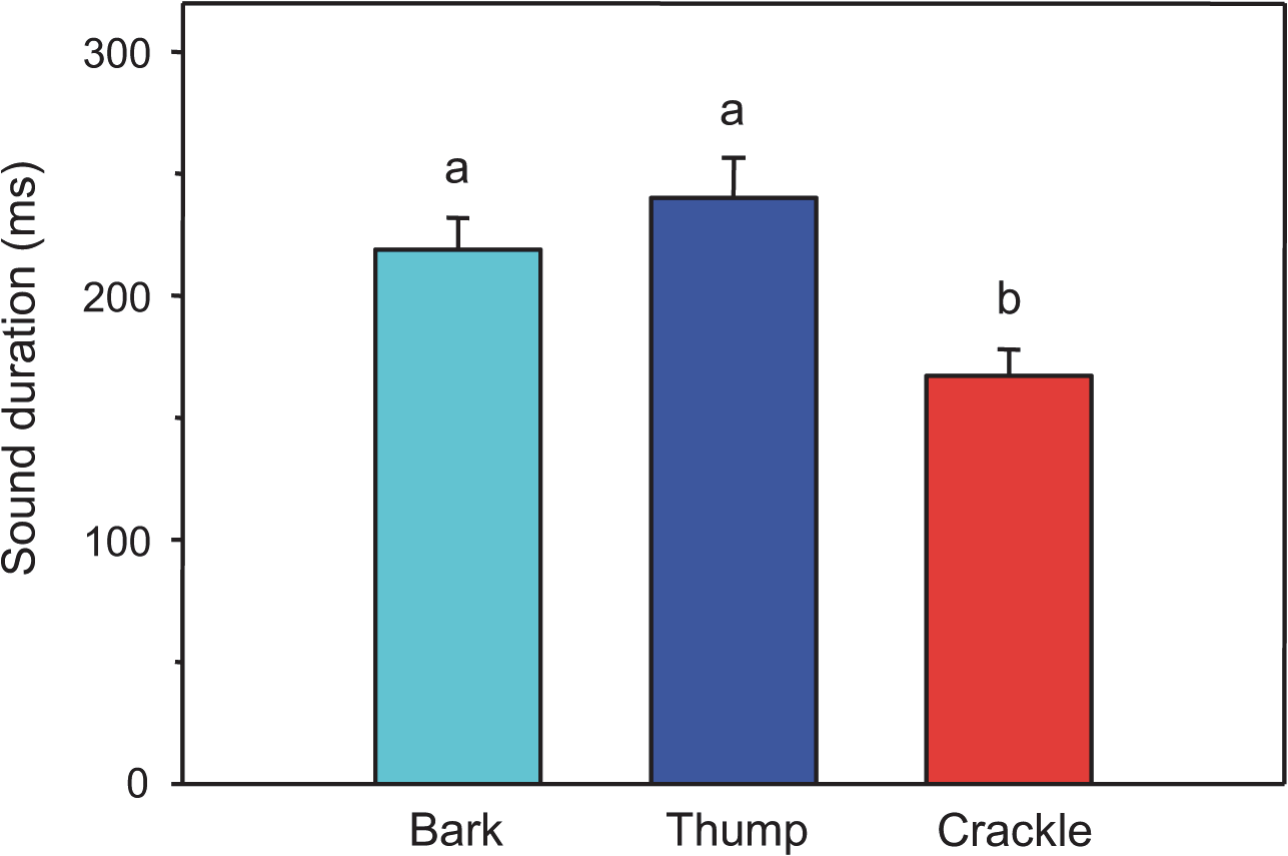
Mean (+ SE) sound duration of male barks and thumps (N = 7) and of female crackles (N = 8). Different letters indicate statistically significant differences between sound types.

**Fig 10:**
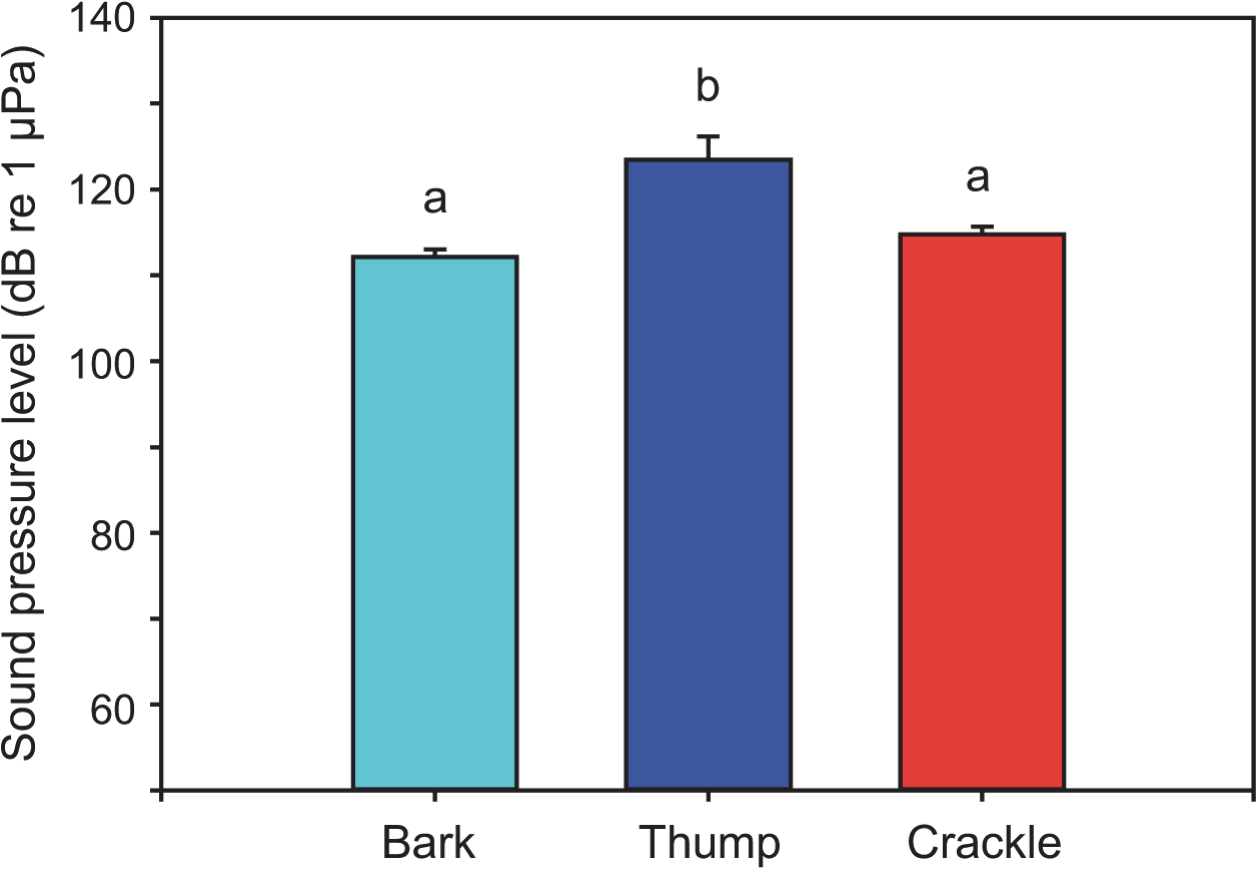
Mean (+ SE) sound pressure level of male barks and thumps (N = 7) and of female crackles (N = 8). Different letters indicate statistically significant differences between sound types.

**Fig 11:**
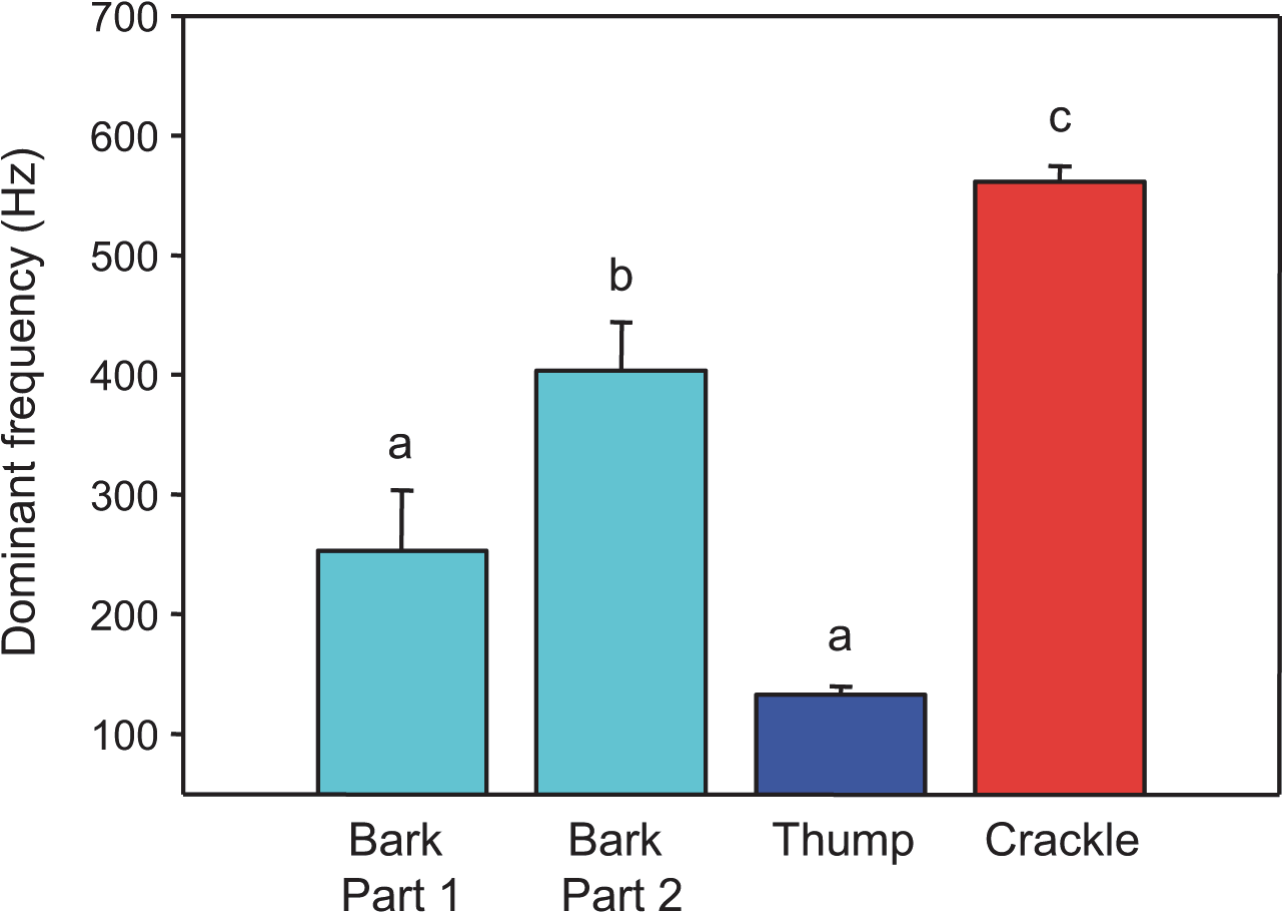
Mean (+ SE) dominant frequency of male barks and thumps (N = 7) and of female crackles (N = 8). Different letters indicate statistically significant differences between sound types.

The sound duration differed between sound types (one-way ANOVA: F_2,19_ = 8.06, p < 0.01). The Bonferroni post-hoc test revealed that barks and thumps were similar in sound duration, but were significantly longer than crackles (Fig. 9). SPLs differed between sound types (one-way ANOVA: F_2,19_ = 11.07, p ≤ 0.001) (Fig. 10). Thumps were significantly louder than barks and crackles, but no such difference was found between barks and crackles (Bonferroni post-hoc test). Furthermore, the dominant frequencies varied between sound types (one-way ANOVA: F_3,25_ = 33.95, p < 0.001) (Fig. 11). Mean dominant frequencies of crackles were much higher than of other sound types. The first part of barks and thumps did not differ significantly in dominant frequency (Bonferroni post-hoc test).

### Correlations between morphological measures and sound characteristics

Sound characteristics of male barks and thumps and female crackles were significantly correlated to morphological measures when males and females were pooled. Duration of male thumps and female crackles combined increased with mean total length and pectoral spine length (Table 2). In contrast, male barks and female crackles were correlated only to pectoral spine length (Table 2).

**Table 2:** Correlations between mean sound characteristics (sound duration, dominant frequency, sound pressure level) of male and female sound types and morphological variables (total length, pectoral spine length, relative pectoral spine length).

Morphologicalvariables	Sound duration		Sound pressure level		Dominant frequency		
Sound types	B + C	T + C	B + C	T + C	BP 1 + C	BP 2 + C	T + C
Total length	0.360	0.773*	- 0.111	0.818*	- 0.435	- 0.401	- 0.446
Pectoral spine length	0.677*	0.842*	- 0.471	0.784*	- 0.885*	- 0.767*	- 0.957*
Relative pectoral spine length	0.667*	0.708*	- 0.473	0.609*	- 0.862*	- 0.741*	- 0.991*

N = 15. B—bark, BP—bark part, C—crackles, T—thumps. Pearson’s correlation coefficients are given. Asterisks indicate statistically significant differences.

Mean SPLs of male thumps and female crackles were positively correlated with total length and the pectoral spine lengths (Table 2). Such a correlation was not found for SPLs of barks and crackles (Table 2).

Interestingly, dominant frequencies of sounds were correlated to pectoral spine length but not to total length (Table 2). Individuals with larger pectoral spines emitted sounds of lower frequency. Mean dominant frequencies of the first part of barks (or the second part of barks), or of thumps and females crackles combined, were negatively correlated to pectoral spine lengths (Table 2).

Thus, mean sound characteristics were always correlated to relative pectoral spine lengths, except for SPLs of male barks. In contrast, only sound duration and SPLs of thumps and crackles combined were correlated to total length (Table 2).

### Auditory sensitivities and comparison to sound spectra

**Fig 12:**
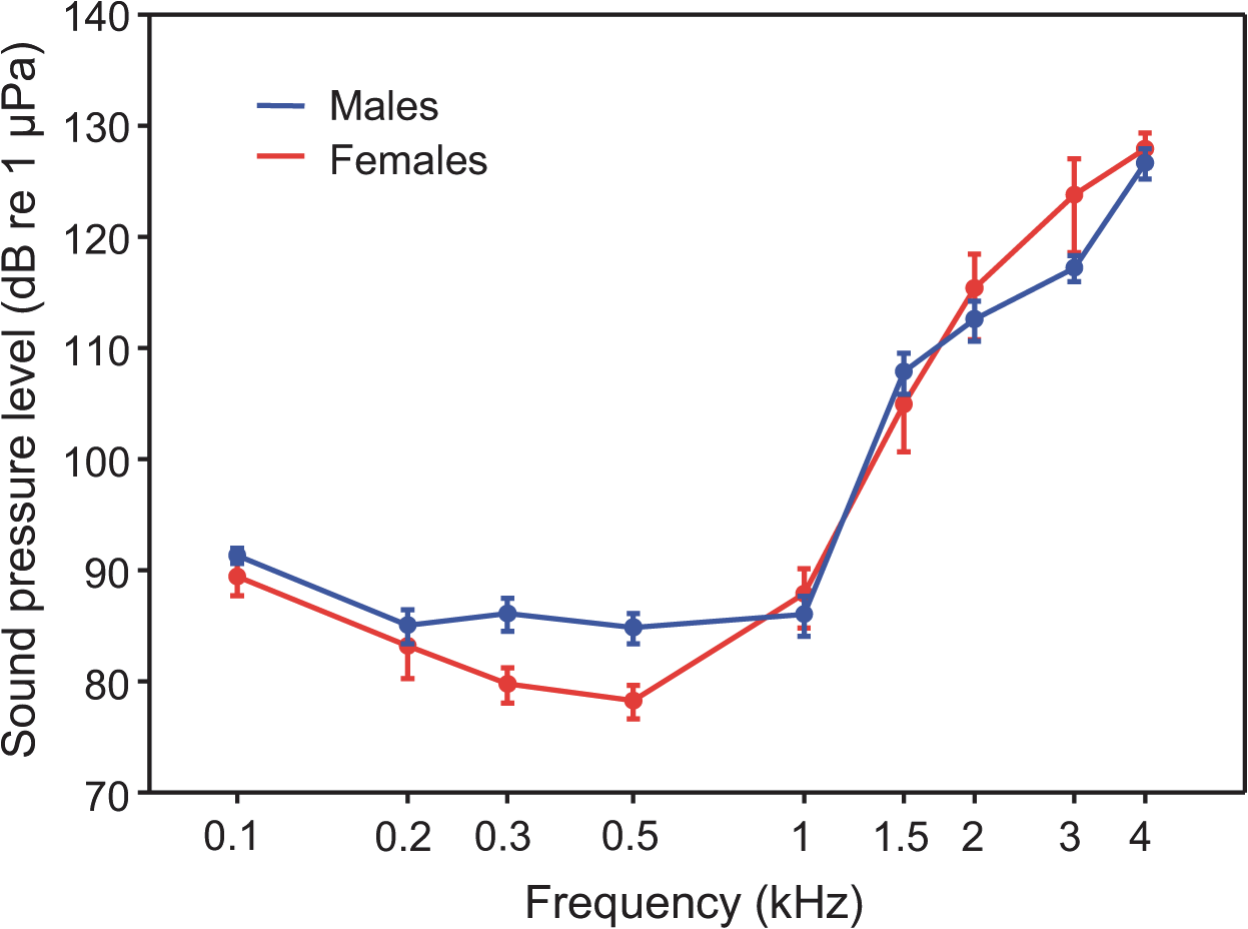
Mean (± SE) auditory sensitivities of male (N = 6) and female (N = 6) *M. thoracata*.

All fish detected tone bursts between 100 Hz and 4 kHz. Hearing curves of both sexes were U-shaped with best auditory sensitivities between 0.2 and 1 kHz (Fig. 12). Hearing abilities decreased rapidly above 1 kHz. Thresholds increased by 41 dB between 1 and 4 kHz in males and females. Overall, hearing thresholds did not differ between sexes (two-way ANOVA: F_8, 89_ = 1.2, n. s.). Females had better hearing between 0.1 and 1 kHz (two-way ANOVA: F _1, 50_ = 7.72, p < 0.01) but not between 1 and 4 kHz (two-way ANOVA: F _1, 49_ = 0.007, n.s.). No significant interaction was found between sex and frequency. Thus, the difference in auditory sensitivity between 0.1 and 1 kHz was not frequency-dependent.

**Fig 13:**
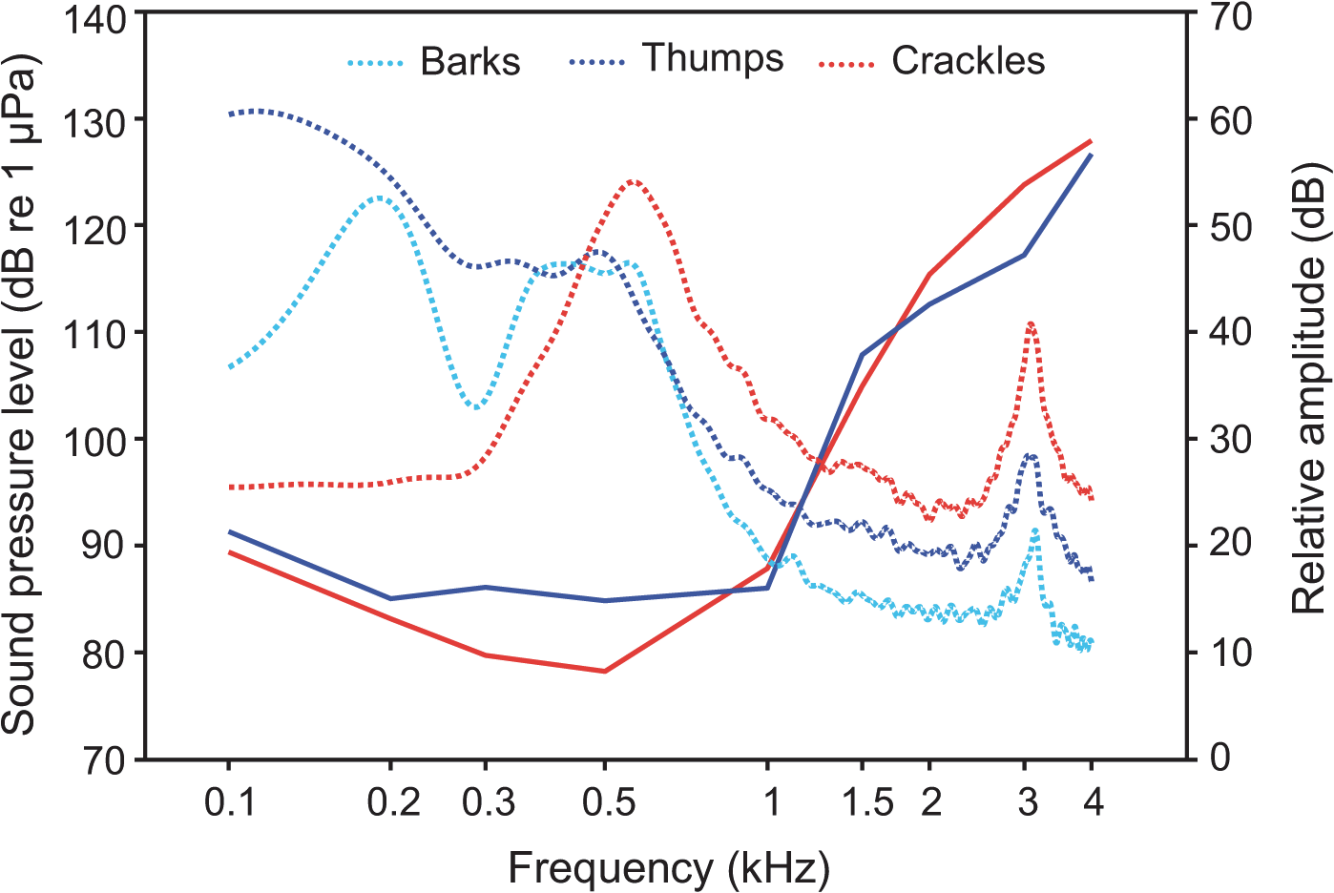
Mean auditory sensitivities of male and female *M. thoracata* (solid lines) in relation to spectral characteristics of sounds (dotted lines). Power spectra of sounds were averaged of sounds of all individuals and are shown in relative amplitude values (right Y-axis).

Both sexes showed best auditory sensitivities in frequency ranges where main energies of sounds were concentrated (Fig. 13). The greatest energy of sounds was concentrated from 180 to 620 Hz in male barks, from 100 to 540 Hz in male thumps and from 470 to 750 Hz in female crackles. Main energies of male and female sounds were much lower than the resonant frequency of the test tank (see energy peak at 3 kHz in Fig. 13).

## Discussion

### Sexual dimorphism and sound-producing mechanism

Male *M. thoracata* possess relatively longer pectoral spines than females. This agrees with the general finding that males typically have larger sonic organs than females [2]. This sexual dimorphism was found in pectoral sonic organs in gouramis (genus *Trichopsis*; [12] and in drumming muscles in toadfish (genus *Opsanus* and *Porichthys*; [14, 15]), cods (genus *Melanogrammus*; [7]), cusk-eels (genus *Ophidion*; [17, 49]) and drums (genus *Argyrosomus*; [8, 16]). However, in the specious order Siluriformes (approximately 3500 species), only species of the family Callichthyidae are known to have sexually dimorphic sonic mechanisms. Males of *C. paleatus* possess relatively longer and thicker pectoral fins than females [13], as well as males of *Dianema urostriatum* and *Callichthys callichthys* [43] which is in accordance with the current finding in *M. thoracata*.

Video analysis revealed that both sexes of *M. thoracata* produced sounds during rapid pectoral fin movements. Therefore, we assume that sound production is based on a stridulatory mechanism, well known in numerous catfish families [50]. While we observed that male barks were produced during abduction of pectoral fins, the movement of pectoral spines during generation of thumps and crackles remains unclear due to faster movements of fins and technical limitations of a standard video recording system. Pruzsinszky and Ladich [13] mentioned that *C. paleatus* produced sounds by abducting the pectoral fins alternately. Similarly, Heyd and Pfeiffer [51] reported that in the callichthyid catfish *D. urostriatum* sounds were produced during abduction, and all members of the family Callichthyidae probably generate sounds during abduction of pectoral fins. We assume that barks and thumps are generated by a single pectoral fin abduction and crackles by several fin abductions depending on the number of elements within a crackle sound.

Interestingly, male barks showed low-frequency harmonic content, indicating the presence of a swimbladder drumming mechanism, similar to many other catfish families [2, 50]. Dissection, however, revealed that *M. thoracata* possesses tiny and paired bony encapsulated bladders, which lack any drumming muscles, a common characteristic of the family Callichthyidae [52]. Kaatz et al. [28] revealed by scanning electron microscopy that members of the subfamily Callichthyinae, such as *Callichthys*, *Dianema*, *Megalechis* and *Hoplosternum*, lack ridges at the dorsal process of the base of the pectoral spine, but possess convolutions instead. These may partly explain the lack of ability to produce (high frequency) “creaking” stridulation sounds [28]. The low-frequency character of *Megalechis* sounds indicates that the pectoral mechanism is highly modified as compared to other catfish species.

### Sexual differences in agonistic behaviour

The current study revealed that agonistic behaviour of male and female *M. thoracata* differed from each other. Male agonistic behaviour mainly consisted of fin displays and jerks, whereas female behaviour was characterized by circling, head nodding, sneaking and striking. Agonistic sounds were produced by males and females in different behavioural contexts. While males uttered thumps during threatening displays and barks mostly during approaching and swimming, females, in contrast, emitted crackles mainly during chasing behaviour. Threatening displays in males consisted of a sequence of visual displays shown by both opponents, during which both may produce sounds. In contrast, chasing behaviour in females was typically shown in one individual, which vocalized when following or moving toward the other fish.

Acoustic signalling by females during agonistic interactions has been described in several families such as cichlids [25], gobiids [53], gouramis [20], sculpins [24], and toadfish [54]. However, sex-specific differences in agonistic behaviour and vocalizations have not been described in any family. Thus, for example, both sexes of the bicolour damselfish *Stegastes partitus* (formerly *Pomacentrus partitus*) produced intense single-pulsed pops during aggressive interactions [55]. In *C. gobio*, both sexes produced knocks and growls while defending their territories, with aggressive calling being mainly size and not sex dependent [24]. Large females were similarly successful as males in defending territories and they produced more sounds than smaller males [19].

Based on the present data, male *M. thoracata* seem to be more aggressive than females as revealed by the much higher number of attacks (40 in males versus four in females) observed during a similar number of same-sex contests. The vocalizing behaviour of *M. thoracata* differs markedly from the closely related callichthyine subfamily member *C. paleatus*, in which only males produced trains of sounds during courtship and in which aggressive behaviour was absent during dyadic contests [13]. Kaatz and Lobel [56] mentioned that male *Corydoras* spp. emitted agonistic chase sound only before, during and after spawning. This difference between the genera *Corydoras* and *Megalechis* is primarily due to their different mating systems. Male *Megalechis* are territorial and defend nest sites, while male *Corydoras* do not build nests or show parental care.

While male thumps and female crackles were emitted only at distances within one to two body lengths, male barks have also been emitted at much larger distances and in the absence of opponents. For this reason, Mayr [27] called male thumps ‘aggressive sounds’ and male barks ‘territorial sounds’. Mayr’s terms have not been used in the current study because they imply unproven functions. Interestingly, the present study showed that barks were emitted only after prior agonistic interactions. Thus, bark production in the absence of intruders might reflect a high level of arousal and less so territorial signalling. We therefore assume that *M. thoracata* does not produce territorial advertisement signals, similar to toadfish or damselfish, which vocalize without prior interactions with conspecifics [55, 57]. Furthermore, our study reveals that both sexes of *M. thoracata* start to defend territories at the age of at least ten months, in contrast to the prior observations by Mayr [27], who claimed that this species exhibits first agonistic behaviours at the age of one and a half years.

The observation that fin beating and sound production occurred at short distances to the opponent indicates that water movement may be detected by the lateral line. This may constitute a third communication channel besides the visual and acoustic ones. Such hydrodynamic cues are potentially involved in all short-distance agonistic and reproductive activities in fishes but seldom unequivocally proven. Satou et al. [58] showed that visual and vibrational (lateral line detected) stimuli elicit spawning behaviour in male red salmon *Oncorhynchus nerka*.

### Sex-specific differences in sound characteristics

Our study revealed three different agonistic sound types (two in males, one in females) based on physical characteristics of sounds. Mean sound duration in *M. thoracata* differed between sexes, with female crackles (167 ms) being shorter than both male barks (219 ms) and thumps (240 ms). Mean SPLs of thumps (123 dB) were higher than those of barks (112 dB) and crackles (115 dB). Mean dominant frequency of female crackles (562 Hz) was much higher than that of male sounds (132–403 Hz). In contrast, Mayr [27] wrote that aggressive signals (= thumps) were shorter than territorial sounds (= barks). Nonetheless, there is a lack of information about whether male sounds differ from female sounds because the sound characteristics have not been compared statistically between sexes. Differences between the current and the prior study by Mayr [27] may reflect that the present study investigated subadult fish and the former only adult reproductive fish. Our fish were most likely immature because we observed no reproductive behaviour in community tanks.

Sound production and sound characteristics of male and female fish have seldom been described and much less compared statistically (for a review see [6]). In the osphronemid *T. vittata*, male croaking sounds were louder, but the temporal and spectral characteristics did not differ [20]. Lagardère et al. [21] reported that female *C. boraborensis* produced longer sound pulses than males. Similarly, Brantley and Bass [54] reported that the duration of agonistic grunts of type II sneaker males in *P. notatus* was shorter than that of females, but this observation was not compared to sounds of territorial type I males. In *O. rochei*, females usually emitted shorter calls that differed from male sounds [17]. Oliveira et al. [26] showed that both sexes of the longsnout seahorse *Hippocampus reidi* produced clicks during feeding and courtship and growls in distress situations when hand held. Males and females were similarly sized and did not differ in most sound characteristics measured (pulse durations, pulse periods, dominant frequencies, sound pressure levels) except one. Similarly to *T. vittata*, male *H. reidi* produced louder courtship clicks than females.

The most common intraspecific variation in fish sound characteristics is in the dominant frequency [1]. Decrease in dominant sound frequencies with increase in body size is a general phenomenon in animals, based largely on resonance [3]. In the present study, differences in morphological measures such as body size and pectoral fin length explain differences in sound characteristics to a certain degree. Sound duration and intensity increased with pectoral spine length, whereas the dominant frequency of sounds decreased. Body size was not correlated with sound features (except for the correlation between male thumps and female crackles versus sound duration and sound level). This indicates that the size of the sound-generating structures, namely the pectoral spine—girdle system, primarily determines sound characteristics. It is assumed that the dominant frequency is mainly determined by the resonance properties of the pectoral mechanism similar to *Ictalurus punctatus* [59]. This agrees with the findings in *C. paleatus*, in which the sound duration of distress calls was positively correlated with the relative pectoral spine length [13]. Similarly, Ladich [20] argued that differences in levels of agonistic sounds of *T. vittata* might reflect the larger pectoral mechanism in males [6, 12]. Lagardere et al. [21] supposed that differences in the duration of the pearlfish *C. boraborensis* sounds might be because females lack a distinct swimbladder bulb (with yet unknown function) at their posterior end which seems to influence sound characteristics. Furthermore, mature female pearlfish are longer than males [60], and differences in sound characteristics may be due to body size differences as well. Kéver et al. [17] demonstrated in the cusk-eel *O. rochei* a tight relationship between the morphology of the sonic apparatus and sound characteristics, with males showing more morphological modifications that may reflect a greater specialization for sound production.

### Sex-specific differences in hearing abilities in fish

Sex-specific differences in hearing have rarely been studied in fish [5, 61]. Investigations on *C. paleatus* [33], *Abudefduf abdominalis* [35] and the Atlantic Molly *Poecilia mexicana* [34] did not reveal any differences between sexes. Overall hearing sensitivities in male and female *M. thoracata* do not differ from each other, but females had better hearing below 1 kHz. Only Zeyl et al. [31] and Maruska et al. [32] described differences in hearing abilities between sexes in fish. Zeyl et al. [31] observed that female round gobies *Neogobius melanostomus* had higher sensitivities than males between 300 and 500 Hz and possessed a higher density of saccular hair cells. Maruska et al. [32] found intersexual differences depending on dominance and reproductive status of males and females of the cichlid *A. burtoni*, but did not compare sexes directly. Subordinate males had lower thresholds than dominant males between 600 and 800 Hz, whereas gravid females had about 5 to 15 dB lower thresholds at low frequencies between 100 to 600 Hz than mouth-brooding females [32]. The authors assumed that higher levels of sex steroids explain these differences. A detailed analysis revealed that female *Megalechis* hear somewhat better in the frequency range between 0.1 and 1 kHz, in particular at 0.3 and 0.5 kHz at their most sensitive frequencies. The auditory sensitivity detected at low frequencies (100–200 Hz) may partly be due to contribution of lateral line neuromasts. Higgs and Radford [62] showed that AEP-thresholds increased by 10–15 dB in the goldfish *Carassius auratus* at 100 and 200 Hz when canal neuromasts were ablated.

**Fig 14:**
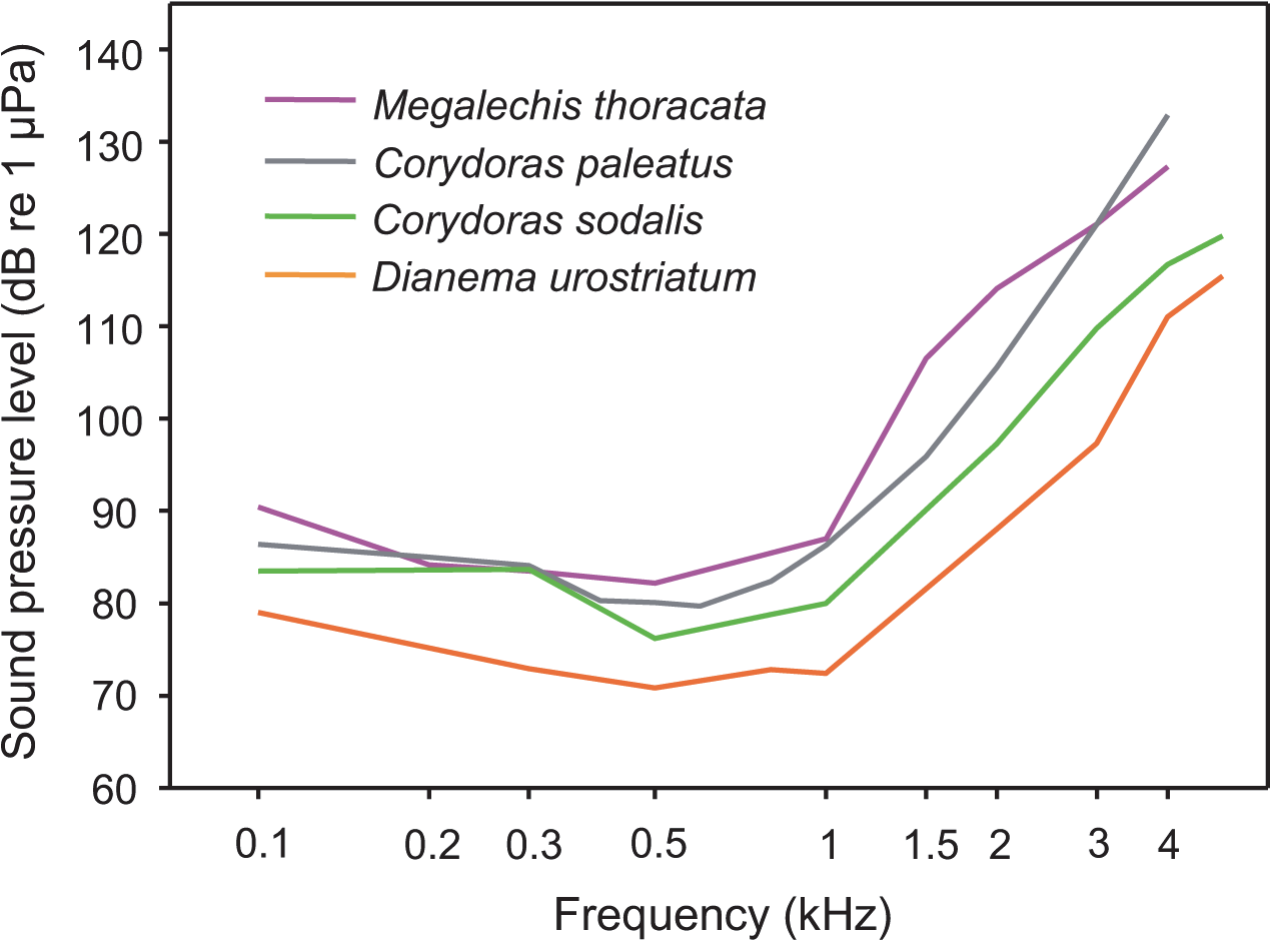
AEP-audiograms of all representatives of the family Callichthyidae investigated in the recent and in prior studies. *Megalechis thoracata* (current study, females and males pooled), *Corydoras paleatus* [33], *Corydoras sodalis* and *Dianema urostriatum* [52].

AEP audiograms of both sexes of *M. thoracata* are very similar to hearing curves of other members of the family Callichthyidae investigated in the same lab (Fig. 12, 14). All show best sensitivity between 0.3 and 1 kHz and a step decrease in sensitivity above 1 kHz [33, 52]. This step decrease is certainly due to the tiny and encapsulated swimbladders in callichthyids in general [52] and *Megalechis* in particular [63]. Lechner and Ladich [52] showed that smaller swimbladders and fewer Weberian ossicles results in a decrease in sensitivity above 1 kilohertz.

### Acoustic communication

In *M. thoracata*, main energies of sounds were found below 1 kHz at the most sensitive frequencies. Both sexes show a similar match between spectral content of sounds and best hearing ability. Based on the results of the present study, male and female *Megalechis* are well adapted for acoustic communication. The relatively low SPL of sounds (mean SPLs: 111–120 dB) indicates that fish communicate at close distances to each other. Studies in several sound-producing taxa revealed a fairly good match between main energies of sounds and the best hearing range [33, 34, 37–41]. Lechner et al. [41] found even a match between the spectral content of sounds and best hearing sensitivity in six different size groups in the mochokid catfish *Synodontis schoutedeni*. Ladich [33] argued that these correlations suggest that sound-producing mechanisms evolved in correlation with hearing abilities in fishes. Similar to the present study, Maruska et al. [35] assumed that the damselfish *A. abdominalis* communicate at close distance, which might help explain the low sound intensity (mean SPLs: 105–130 dB). In contrast to the current data, Ladich [33] revealed a mismatch in the closely related *C. paleatus*, in which the hearing ability decreased rapidly above 800 Hz, and the main energies of sounds were concentrated between 1 and 2 kHz.

In summary, this is the first investigation showing sex-specific differences in agonistic behaviour, sound production, sound characteristics and hearing abilities using same-sex agonistic contests. The data reveal clear differences in agonistic behaviour, which have not been shown in any fish species before. This study furthermore indicates that differences in sound characteristics between sexes seem to be due to the dimorphism in the pectoral sonic organs in *Megalechis*.
